# Endolysosomal pathway activity protects cells from neurotoxic TDP-43

**DOI:** 10.15698/mic2018.04.627

**Published:** 2018-03-21

**Authors:** Christine Leibiger, Jana Deisel, Andreas Aufschnaiter, Stefanie Ambros, Maria Tereshchenko, Bert M. Verheijen, Sabrina Büttner, Ralf J. Braun

**Affiliations:** 1 Institute of Cell Biology, University of Bayreuth, Universitätsstraße 30, 95447 Bayreuth, Germany.; 2 Institute of Molecular Biosciences, University of Graz, Humboldtstraße 50, 8010 Graz, Austria.; 3 Department of Translational Neuroscience, Brain Center Rudolf Magnus, University Medical Center Utrecht, Universiteitsweg 100, 3584 CG Utrecht, The Netherlands.; 4 Department of Neurology and Neurosurgery, Brain Center Rudolf Magnus, University Medical Center Utrecht, Heidelberglaan 100, 3508 GA Utrecht, The Netherlands.; 5 Department of Molecular Biosciences, The Wenner Gren Institute, Stockholm University, S-106 91 Stockholm, Sweden.

**Keywords:** motor neuron disease, amyotrophic lateral sclerosis, frontotemporal dementia, TDP-43, protein aggregation, proteolysis, endolysosomal pathway, endosomal-vacuolar pathway, endocytosis, vacuole, lysosomes, autophagy, cell death, Saccharomyces cerevisiae

## Abstract

The accumulation of protein aggregates in neurons is a typical pathological hallmark of the motor neuron disease amyotrophic lateral sclerosis (ALS) and of frontotemporal dementia (FTD). In many cases, these aggregates are composed of the 43 kDa TAR DNA-binding protein (TDP 43). Using a yeast model for TDP 43 proteinopathies, we observed that the vacuole (the yeast equivalent of lysosomes) markedly contributed to the degradation of TDP 43. This clearance occurred via TDP 43-containing vesicles fusing with the vacuole through the concerted action of the endosomal-vacuolar (or endolysosomal) pathway and autophagy. In line with its dominant role in the clearance of TDP 43, endosomal-vacuolar pathway activity protected cells from the detrimental effects of TDP 43. In contrast, enhanced autophagy contributed to TDP 43 cytotoxicity, despite being involved in TDP 43 degradation. TDP 43’s interference with endosomal-vacuolar pathway activity may have two deleterious consequences. First, it interferes with its own degradation via this pathway, resulting in TDP 43 accumulation. Second, it affects vacuolar proteolytic activity, which requires endosomal-vacuolar trafficking. We speculate that the latter contributes to aberrant autophagy. In sum, we propose that ameliorating endolysosomal pathway activity enhances cell survival in TDP 43-associated diseases.

Our study highlights the pivotal role of endolysosomal pathway activity in TDP 43 proteinopathies. Whereas previous studies focused on the role of autophagy in the clearance of cytotoxic TDP 43, we propose that the endolysosomal pathway is more important than autophagy for the survival of cells accumulating detrimental TDP 43. In both pathways, protein substrates (such as TDP 43) are sequestered into vesicles, either multivesicular bodies (MVBs) or autophagosomes (Figure 1). These vesicles fuse with the lysosomal membrane and subsequently release their content into the lysosomal lumen, where protein degradation occurs via proteases such as Pep4/Cathepsin D. Both vesicular pathways are interconnected. In mammalian cells (but not in yeast), some MVBs fuse with autophagosomes, leading to the formation of amphisomes which then fuse with lysosomes. Moreover, the endolysosomal pathway is required for directing important lysosomal proteases, thus decisively contributing to proteolytic capacity of this organelle.

**Figure 1:**
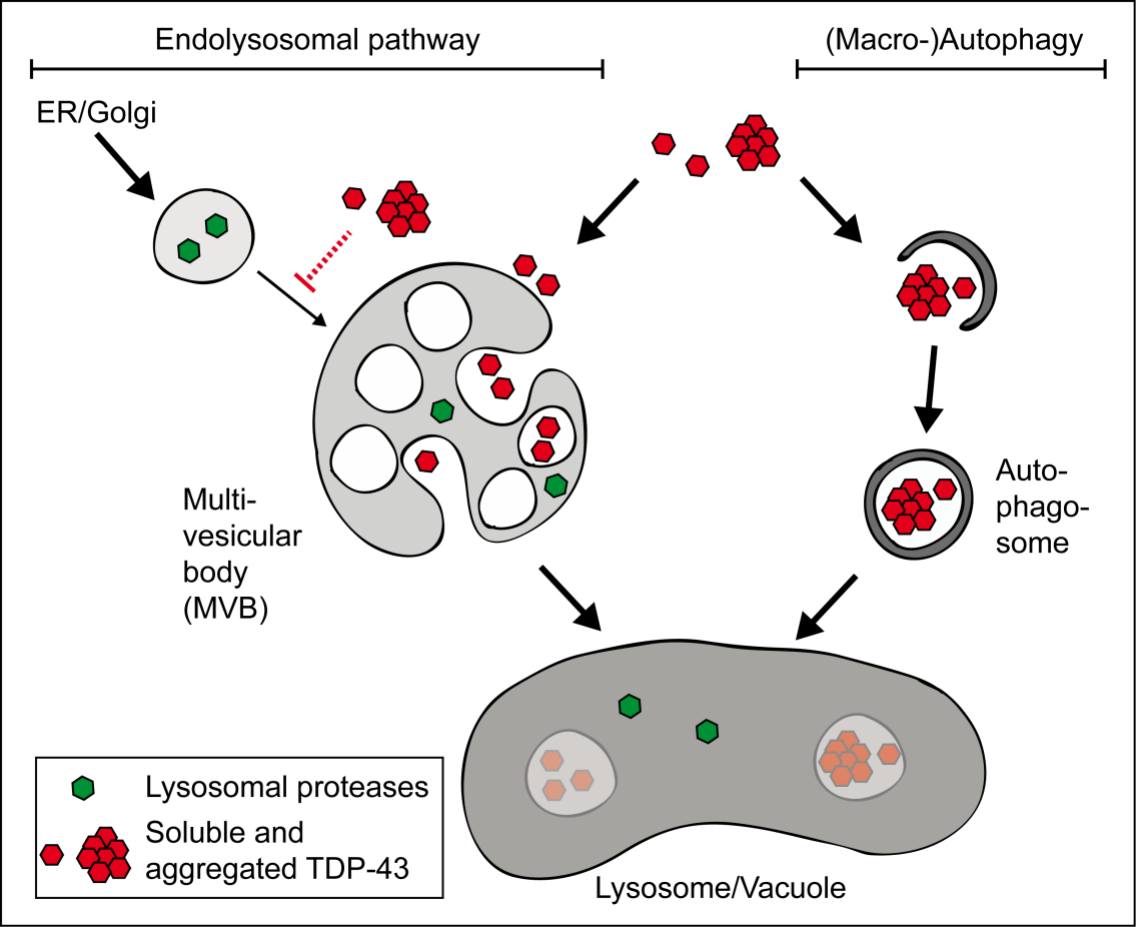
FIGURE 1: Lysosomal degradation of TDP 43. The lysosome (or the yeast vacuole) contains a number of proteases, which enable the degradation of proteins. Protein substrates are transported to the lysosome either via the endolysosomal (or endosomal-vacuolar) pathway (left) or via (macro-)autophagy (right). Both pathways contribute to the degradation of TDP 43. Furthermore, TDP 43 is a potent inhibitor of the endolysosomal pathway, which is required for lysosomal function. For details see main text.

Our study raises a number of questions regarding TDP 43 degradation and cytotoxicity. For instance, in cells accumulating cytoplasmic TDP 43, different types of potentially cytotoxic TDP 43 species can be expected, e.g., monomers, oligomers, and different classes of protein aggregates.

Which kinds of TDP 43 species are recruited into MVBs for degradation via the endosomal-vacuolar pathway, and which kinds of TDP 43 species are sequestered into Atg8-labelled cellular compartments for elimination via autophagy?

For steric reasons, one would expect that MVBs contain smaller TDP 43 species than autophagosomes, which could easily enclose large protein aggregates. It is possible that the endosomal-vacuolar pathway is involved in the clearance of more cytotoxic species than autophagy. This could explain why loss of this pathway is more detrimental for TDP 43-expressing cells than loss of autophagy.

What is the molecular machinery that determines whether TDP 43 is degraded via the endosomal-vacuolar pathway or via autophagy (or via the ubiquitin-proteasome system)?

It is tempting to speculate that the ubiquitylation pattern of TDP 43 plays an important role. Lysine 63 (K63) polyubiquitin chains could favor the endosomal-vacuolar pathway, while K48 polyubiquitin chains could facilitate degradation via the proteasome, and both chains could be substrates for ubiquitin-dependent autophagy.

What is the molecular machinery that recruits TDP 43 into MVBs for degradation via the endosomal-vacuolar pathway?

Whereas there is a significant amount of information regarding the molecular machinery involved in recruiting protein aggregates into autophagosomes, the mechanisms that enable the recruitment of cytosolic proteins into MVBs remain largely unknown.

Autophagy has been shown to be a cytoprotective mechanism to eliminate deleterious protein aggregates. Therefore, pharmacological induction of autophagy has been proposed to be an efficient mechanism to prevent neuronal loss in neurodegenerative disorders. However, this approach was not successful in our yeast TDP 43 model. In contrast, it increased cytotoxicity.

Does the observed inhibitory effect of TDP 43 on endosomal-vacuolar pathway activity and vacuolar proteolytic activity reprogram (normally cytoprotective) autophagy towards cytotoxicity?

If this holds true, a therapeutic approach that addresses both endosomal-vacuolar pathway activity and autophagic turnover should be much more effective than targeting autophagy alone.

Resolving these questions in yeast TDP 43 proteinopathy models and their validations in higher model systems (e.g., worm, fly, murine or patient-induced pluripotent stem cell-derived neuronal models) will improve our understanding of the molecular mechanisms of devastating TDP 43-associated human disorders.

